# Semi-Polycrystalline Polyaniline-Activated Carbon Composite for Supercapacitor Application

**DOI:** 10.3390/molecules28041520

**Published:** 2023-02-04

**Authors:** Neelima Mahato, T. V. M. Sreekanth, Kisoo Yoo, Jonghoon Kim

**Affiliations:** 1 Energy Storage and Conversion Laboratory, Department of Electrical Engineering, Chungnam National University, Daejeon 34134, Republic of Korea; 2 Department of Mechanical Energy, Yeungnam University, Gyeongsangbukdo, Gyeongsansi 38541, Republic of Korea

**Keywords:** polyaniline, semi-polycrystalline polyaniline, activated carbon, electroactive material, conductive polymer–carbon composite, pseudocapacitor, galvanostatic charge–discharge (GCD)

## Abstract

We report on the synthesis of activated carbon-semi-polycrystalline polyaniline (SPani-AC) composite material using in-situ oxidative polymerization of aniline on the carbon surface in an aqueous HCl medium at an elevated temperature of 60 °C. The electroactive polymeric composite material exhibits a uniformly distributed spindle-shaped morphology in scanning electron microscopy (SEM) and well-defined crystallographic lattices in the high-resolution transmission electron microscopy (TEM) images. The X-ray diffraction (XRD) spectrum reveals sharp peaks characteristic of crystalline polyaniline. The characteristic chemical properties of polyaniline are recorded using laser Raman spectroscopy. The cyclic voltammetry curves exhibit features of surface-redox pseudocapacitance. The specific capacitance calculated for the material is 507 F g^−1^ at the scan rate of 10 mV s^−1^. The symmetrical two-electrodes device exhibits a specific capacitance of 45 F g^−1^ at a current density of 5 A g^−1^. The capacitive retention calculated was found to be 96% up to 4500 continuous charge–discharge cycles and observed to be gradually declining at the end of 10,000 cycles. On the other hand, Coulombic efficiency was observed to be retained up to 85% until 4500 continuous charge–discharge cycles which declines up to 72% at the end of 10,000 cycles. The article also presents a detailed description of material synthesis, the formation of polyaniline (Pani) chains, and the role of material architecture in the performance as surface redox supercapacitor electrode.

## 1. Introduction

Supercapacitors exhibit great energy storage properties and fine applications in several portable devices, e.g., solar energy, watches, electrical vehicles, etc. [1,2,3,4]. The charge storage in an electroactive material occurs mainly through three mechanisms, viz., (a) EDLC mechanism or electrical double layer capacitor mechanism essentially through a non-Faradaic process in which the electrical energy occurs via ionic charge separation at the electrode electrolyte interface. 

In this process, no change in phase of the material is involved [5,6]; (b) redox capacitor mechanism or via Faradaic process in which redox reactions involve change in phase of the material, e.g., metal oxides [5,6,7,8]; (c) ion-insertion mechanism or using a diffusion-controlled process in which charge storage takes place via intercalation/de-intercalation of cations (e.g., Na^+^, K^+^ and H^+^) in the bulk of the electroactive material and follows a slow kinetics [9,10,11,12]. In this category, aq. NiMH rechargeable batteries and non-aqueous Li-ion batteries present good examples. These materials exhibit charge storage through intercalation/deintercalation of H^+^ or Li^+^ ions within their crystalline and layered material architecture, and the entire kinetic process is controlled by the diffusion of cations [6,7,13]. According to Conway definitions, the materials exhibiting the EDLC mechanism of charge storage are called capacitors, and the materials undergoing the redox reaction with phase change of materials are called battery-type materials. Pseudocapacitor materials exhibit properties of both, charge storage occurring via the pure capacitive process as well as via the redox-type Faradaic process. Conductive polymers, such as polyaniline, exhibit pseudocapacitive behavior and deliver high capacitance compared to other organic material-based electroactive materials. Conducting polymers display sharp redox peaks as well as large peak separation in cyclic voltammetry curves characteristic of battery material [5,6,14,15]. Conducting polymers, by virtue of their extended conjugation (alternate double bonds) through the overlapping of unhybridized pπ-orbitals, offer unusual electronic properties, e.g., electrical conductivity, electron transition, low ionization potential, and high electron affinity [2,16,17]. Furthermore, conducting polymers exhibit unique properties in terms of redox reactions, and yet undergo no phase change during the charging–discharging processes, and such materials are termed ‘redox capacitors’ [14]. Reports on the synthesis of novel semi-polycrystalline polyaniline, enhanced electrochemical behavior, and the mechanism of charge storage in antibonding pπ-molecular orbitals can also be found in the references [18,19,20].

Polyaniline is one of the most extensively researched conductive polymers, well known for its non-toxicity, environmental compatibility, biodegradability, appreciable electronic properties, multiple redox states, convenient in doping with a variety of dopant materials ranging from organic and inorganic redox-active materials to inert carbon materials, and ease in synthesis, either electrochemically, or via chemical oxidative polymerization in a standalone aqueous acidic environment, or by using surfactant molecules, such as para-phenylene diamine, sulfuric acid, *p*-toluene sulfonic acid, camphor-10-sulfonic acid, and so on [3,6,21]. However, it has also been reported that conducting polymers, despite exhibiting appreciably good specific capacitances, display a lower cycle life in their charge–discharge performances compared to other carbon-based materials when employed as an electrode. The latter is attributed to the relatively lower stability of redox sites in the polymer architecture for many repeated redox processes [22]. In a quest to improve the electrochemical properties of the polyaniline, doping of the material has shown promising results. However, the introduction of dopants in the material also introduces characteristic influences in the morphology and the overall architecture of the base polymeric material. Here, nature, size, amount, and presence of functional groups in the dopant molecule play crucial roles in the development of the overall final composite architecture and its electrochemical properties. Different dopant molecules bear different functional groups which possess different molecular sizes and give rise to different pore sizes in the product composite morphologies post-synthesis and consequently influence the overall electrochemical behavior. The morphology of the conductive polymers can also be altered by modifying the reaction parameters during the synthesis stage, e.g., change of temperature and/or application of pressure (or hydrothermal treatment) [21,23,24].

Carbon materials are known for their structural and chemical stability, cyclic stability, large surface area, abundant availability, and low cost [2,25]. Although the carbon materials possess a large surface area, they have been reported to exhibit relatively lower capacitances. The latter can be improved by compositing carbon materials with conducting polymeric materials [19,20]. In this study, an activated carbon-polyaniline composite was synthesized via chemical oxidative polymerization of aniline in situ at an elevated temperature of 60 °C, and its electrochemical properties were investigated. In the high-resolution TEM images, the material exhibits randomly scattered nanostructured patches with well-defined crystalline lattice planes and sharp peaks in the X-ray diffraction spectrum characteristic of a crystalline material. There have been multiple reports on polyaniline-activated carbon composite supercapacitor applications; however, the synthesis route discussed in the present article with a unique feature in the final product (i.e., appearance of crystallographic phases in the composite matrix) has not been reported previously by any other researcher. The development of an alternate theory explaining the mechanism of charge storage mechanism (increase in the specific capacitance with an increase in the current density in a three-electrode as well as two-electrode device set-up) occurring in the composite matrix employing quantum chemistry is also noteworthy.

## 2. Results

### 2.1. Microstructure, Phase, and Chemical Properties of SPani-AC

**Figure 1:**
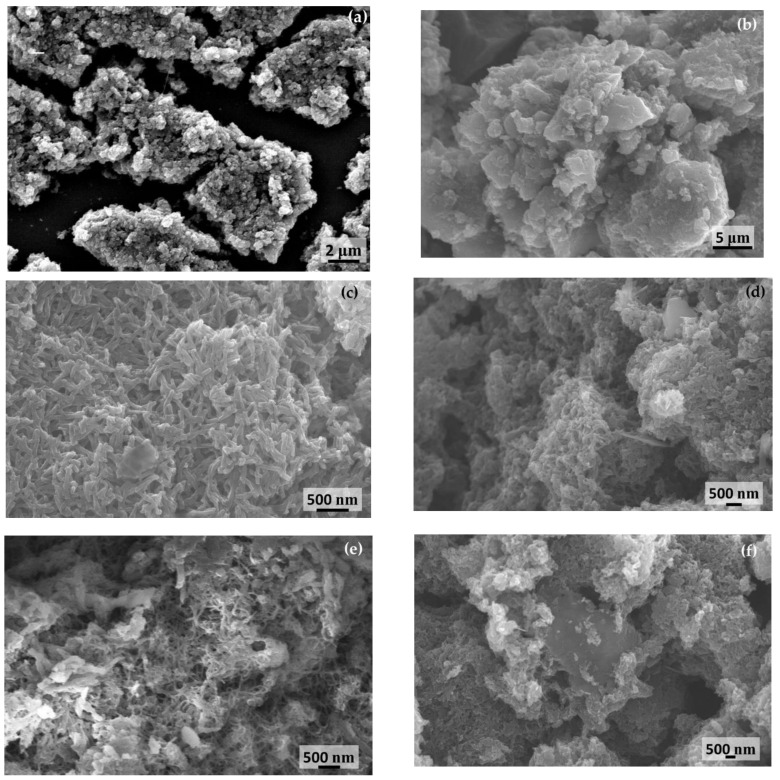
Scanning electron micrographs of (**a**,**b**) activated carbon (AC); (**d**–**f**) semi-polycrystalline polyaniline-activated carbon composite (SPani-AC).

The scanning electron micrographs of activated carbon and SPani-AC composite is shown in Figure 1a–f. 

**Figure 2:**
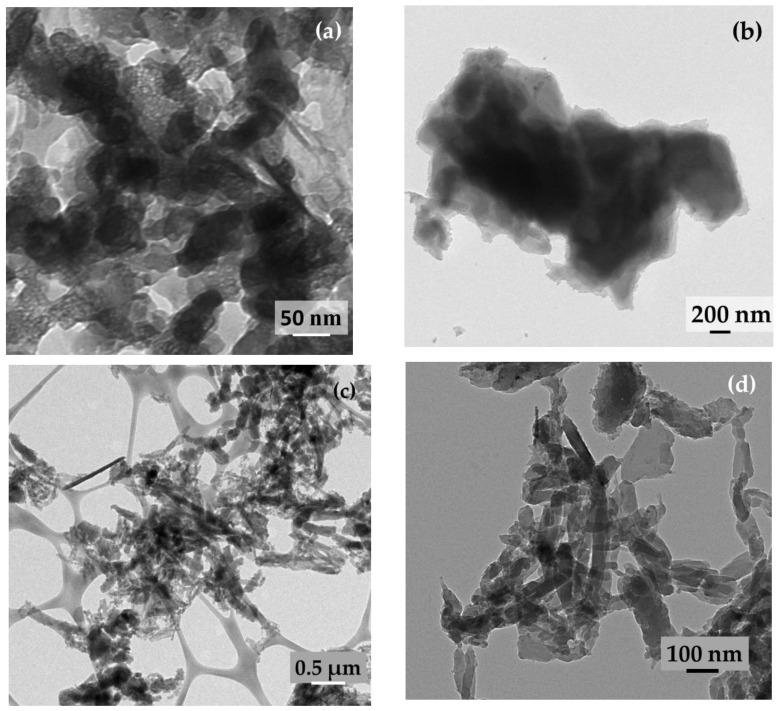

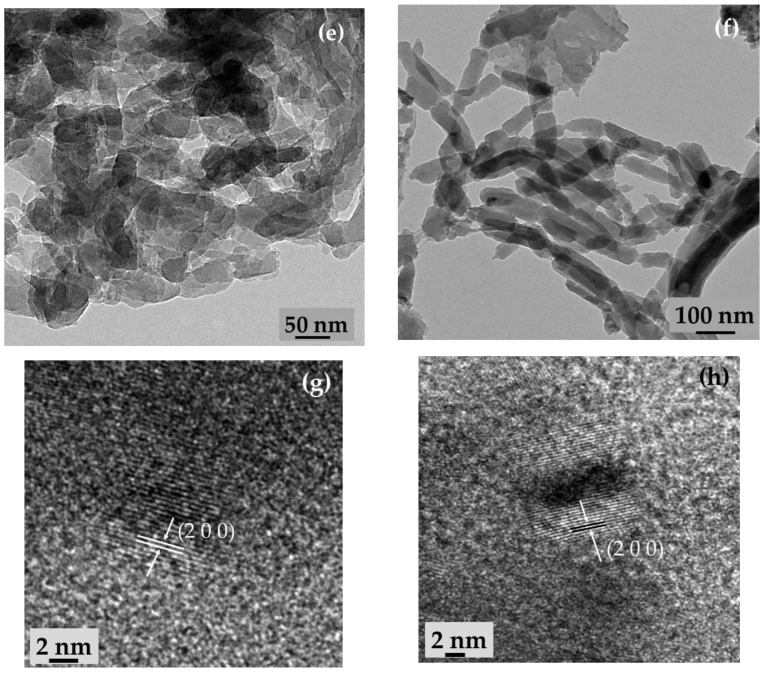
TEM images of (**a**,**b**) activated carbon particles; (**c**–**f**) semi-polycrystalline polyaniline-activated carbon (SPani-AC) composite; (**g**,**h**) high-resolution transmission electron (HR-TEM) micrographs of SPani-AC composite showing crystallographic phases.

The activated carbon appears to be a porous mass, and polyaniline appears to be flaky spindle- or fibril-shaped entangling carbon particle agglomerates. The flakes appear to possess clean boundaries and not fused with one another. In transmission electron micrographs (Figure 2a–f), the activated carbon particles appear as irregularly shaped entities; polyaniline appear as long fibrillar structures and randomly scattered; SPani-AC appear as polyaniline fibers entangling tiny carbon particles in its folds. In high-resolution TEM images, the material appears to be semi-polycrystalline with randomly scattered nanostructured crystalline patches exhibiting well-defined crystallographic lattices. The lattices corresponding to (2 0 0) the crystallographic plane are shown in Figure 2g,h. 

**Figure 3:**
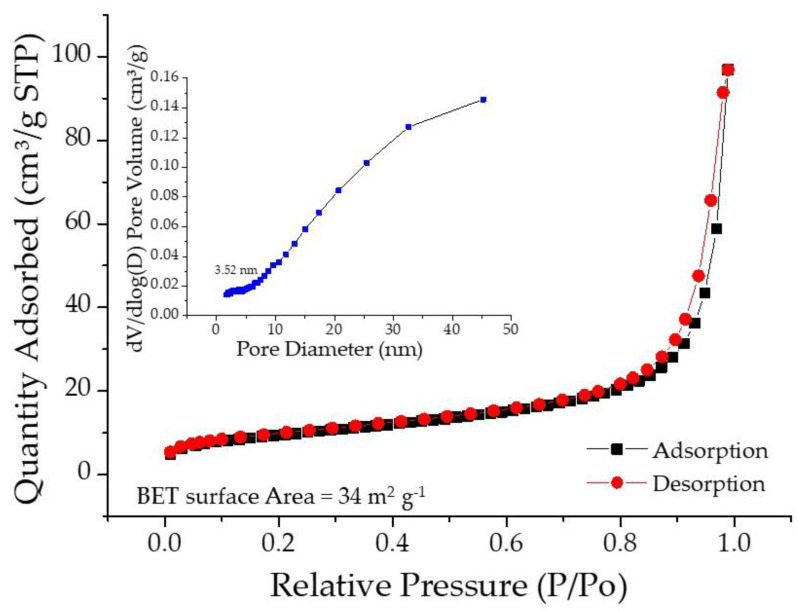
Nitrogen adsorption–desorption curves with the corresponding pore size distribution plots for the composite material SPani-AC.

A large surface area with open pore-structures in the material micro-architecture is crucial for high performance in terms of capacitive charge storage. In this view, the nitrogen adsorption–desorption curve and the corresponding pore size distribution plot for SPani-AC composite is shown in Figure 3. The activated carbon used in this study was a commercial product, mesoporous in nature (pore size in the range 2–4 nm) with a BET surface area of 162 m^2^ g^−1^ (noted from the product specification provided by the company). The BET analysis of the SPani-AC composite material reveals a surface area of 34 m^2^ g^−1^, mesoporous in nature (pore size distribution in the range of 2–4 nm). The curve shows an increasing trend beyond a 6 nm pore diameter and a subject for additional investigation. 

**Figure 4:**
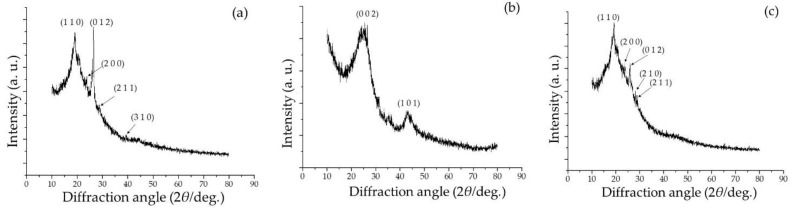
XRD spectra of (**a**) standalone semi-polycrystalline polyaniline (SPani), (**b**) activated carbon (AC), and (**c**) semi-polycrystalline polyaniline-AC (SPani-AC) composite.

The composite material hence obtained by synthesis at an elevated temperature is justifiably termed semi-polycrystalline-activated carbon composite (SPani-AC). X-ray diffraction spectra of standalone semi-polycrystalline polyaniline (SPani), activated carbon, and SPani-AC composite are shown in Figure 4a–c. 

**Figure 5:**
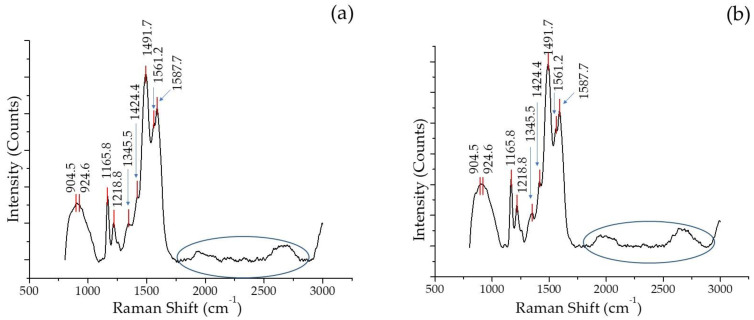
Laser Raman spectra of the (**a**) SPani and (**b**) SPani-AC composite.

The spectrum corresponding to SPani and SPani-AC display sharp peaks indicative of the crystalline character of the material. Non-crystalline and nanostructured materials display broad hump-like features in the XRD spectra. The peaks in the spectra of SPani and SPani-AC composite at 2*θ* diffraction angles of 19.2°, 24.0°, 25.8°, 28.9°, 29.2°, 32.4°, and 39.5° are assigned to the crystallographic planes, viz., (1 1 0), (2 0 0), (0 1 2), (2 1 0), (2 1 1), (1 2 0), and (3 1 0) according to JCPDS card no. 53-1890. The broad hump-like peaks in the spectrum of activated carbon appearing in the 2*θ* diffraction angle ranges of 15–30° and 40–50° are assigned to the crystallographic planes, viz., C (0 0 2) and C (1 0 1) crystallographic planes associated with the axis of the graphite structure [26]. The peak at 25.8° corresponding to the crystallographic plane (0 1 2) appears to be diminished in the spectrum of SPani-AC (compared to that in the spectrum of SPani), attributed to be arising due to the presence of carbon particles in the composite structure and believed to be resulting from reduction in the number of (0 1 2) crystallographic planes in the composite material upon introduction of the activated carbon. Noticeably, there is no significant difference in the HR-TEM images and XRD patterns of SPani and SPani-AC, suggesting that the inclusion of activated carbon particles in the composite does not significantly affect the microstructure and phases of the SPani. The laser Raman spectra of the standalone Pani and SPani-AC composites are displayed in Figure 5a,b.

The spectra display peaks characteristic of Pani, viz., the bands located at 1588 cm^−1^ are assigned to C-C single bond and double bond stretching in the quinine type ring. The bands appearing at 1345 cm^−1^ and 1491 cm^−1^ correspond to C-N^+^ and C=N stretching associated with charge localization. The band appearing at 1218 cm^−1^, 1340 cm^−1^, and 1491 cm^−1^ are assigned to C-N stretching of amine sites, stretching vibrations of an intermediate bond C-N^+^, and specific C=N stretching modes, respectively. A relatively small bond appearing at 1561 cm^−1^ is assigned to N-H bending vibrations, and a band appearing at 1166 cm^−1^ is assigned to C-H in plane deformation mode associated with quinonoid [27,28,29,30,31,32,33]. It is further added that the product formed in the present study does not contain oligomeric aniline. Throughout the synthesis process, the pH of the solution was close to 1.0 [34]. 

### 2.2. Electrochemical Capacitor Investigation

#### 2.2.1. Cyclic Voltammetry 

**Figure 6:**
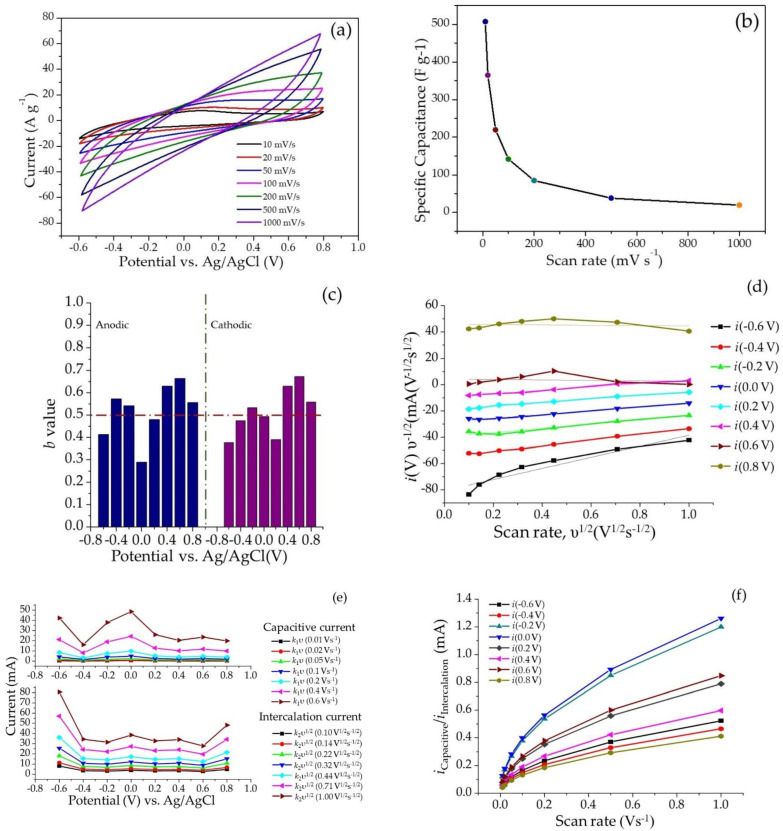
Electrochemical performance of semi-polycrystalline polyaniline-activated carbon (SPani-AC) composite in aqueous 1 N Na_2_SO_4_ electrolyte: (**a**) cyclic voltammograms recorded at different scan rates ranging from 10–1000 mV s^−1^; (**b**) evolution of specific capacitance with respect to different scan rates; (**c**) ‘*b*’ value determination from peak anodic and cathodic currents (log *i_p_* vs. log scan rate); (**d**) linear fit plots of υ^1/2^ vs. *i*(*V*)υ^1/2^; (**e**) variation of capacitive and intercalation currents as a function of potential; (**f**) variation of the ratio of capacitive and intercalation currents with scan rate.

The current responses vs. potential or cyclic voltammograms recorded in the working potential range of −0.6 V to 0.8 V at different scan rates ranging between 10 mVs^−1^ and 1 Vs^−1^ in aqueous Na_2_SO_4_ (1 N) as electrolyte are displayed as overlay plots in Figure 6a. The evolution of specific capacitances is displayed in Figure 6b. 

The specific capacitance ‘*C_s_*’ for the SPani-AC composite electroactive material was calculated by using Equation (1) [35]

(1) $$C_{s}=\frac{\int_{Vi}^{Vf}i.dV}{2m\upsilon \left(Vf-Vi\right)}$$

where ‘*i*’ is the voltametric current (A), ‘*m*’ is the mass of the electroactive material, ‘*v*’ is the voltage scan rate, and ‘(*V_f_ − V_i_*)’ is the applied voltage (*V*). The specific capacitance calculated for the material is 507 F g^−1^ at the scan rate of 10 mV s^−1^, which still maintained a value of ~142 F g^−1^ at a faster scan rate of 100 mV s^−1^ and declines to 19.4 F g^−1^ at a scan rate of 1 V s^−1^. 

#### 2.2.2. Determination of ‘b’ Values

At a particular potential, the current ‘*i*’ and scan rate ‘*v*’ are related as follows (Power law):

(2) $$i\left(V\right)=a\upsilon^{b}$$

The ‘*b*’ value can be calculated from the slope of the log(*i*) vs. log (*v*). The ‘*b*’ values calculated from the peak anodic and cathodic currents (log *i_p_* vs. log scan rate) are shown in Figure 6c. It is possible to determine quantitatively the exact share of the two processes, viz., the double layer charge storage with pseudocapacitance and a diffusion-controlled/ion-intercalation process, using the following equations:

(3) $$i\left(V\right)=i_{Capacitive}+i_{Diffusion\;controlled/ion-intercalation}$$

(4) $$\mathrm{or},\;i\left(V\right)=k_{1}\upsilon +k_{2}\upsilon^{1/2}$$

(5) $$\mathrm{or},\;i\left(V\right)=k_{1}\upsilon^{1/2}+k_{2}$$

Linear fit plots of *i/υ^1/2^* vs. *υ^1/2^* yielding the slope *k_1_* and a *y*-axis intercept *k_2_* values are shown in Figure 6d. Following this, the capacitive current (*k_1_ υ*) and diffusion-controlled/ion-intercalation current (k_2_ υ^1/2^) values can be easily computed (Figure 6e). The variation of capacitive and diffusion/ion-intercalation currents with potential is shown in Figure 6f. It appears that the intercalation of ions led by the diffusion processes is a predominant process occurring in the present case over the capacitive charge–discharge storage process at the two ends of the working potential range, i.e., (−0.6 V) and (0.8 V). The capacitive charge storage mechanism is dominant in the potential range of (−0.2 V) to (0.2 V) as shown in Figure 6e. Further, the pattern of the curves displaying the ratio of the capacitive current and intercalation current increases with an increase in the scan rates. At higher scan rates, the capacitive charge storage mechanism dominates over the intercalation charge storage mechanism. 

#### 2.2.3. Gravimetric Charge–Discharge

**Figure 7:**
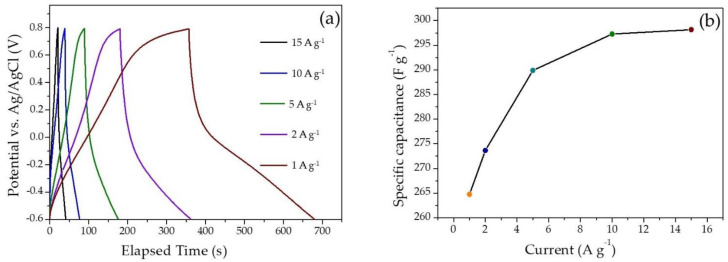
Electrochemical performance of the semi-polycrystalline polyaniline in aqueous electrolyte: (**a**) galvanostatic charge–discharge curves recorded at different current densities ranging from 1.0–15 A g^−1^; (**b**) evolution of the specific capacitance with respect to the different current densities.

The specific capacitances from the gravimetric charge–discharge (GCD) recorded in the working potential range of −0.6 V to 0.8 V by varying the current density from 1 A g^−1^ to 15 A g^−1^, and the variation of the specific capacitance with the current density, are shown in Figure 7a,b. 

The specific capacitance from the GCD curves for a unit mass of the electroactive material and the Coulombic efficiency are calculated from the below equations:

(6) CF g−1=(i×△t)(△V×m)

where, ‘*i*’ is the applied current (A), ‘Δ*t*’ is the discharging time, and ‘Δ*V*’ is the working potential window. For systems displaying non-linear behavior in the GCD curves (Figure 7a) due to pseudocapacitance, an integration formula (Equation (7)) is used instead of Equation (6).

(7) $$C_{F\;g^{-1}}=i\int^{\;}1/V\left(t\right)\;dt$$

The variation of the specific capacitance from the GCD curves with a variation in current density is shown in Figure 7b. The specific capacitance calculated was 298.2 F g^−1^ at 15 A g^−1^. 

#### 2.2.4. Electrochemical Impedance Spectroscopy

**Figure 8:**
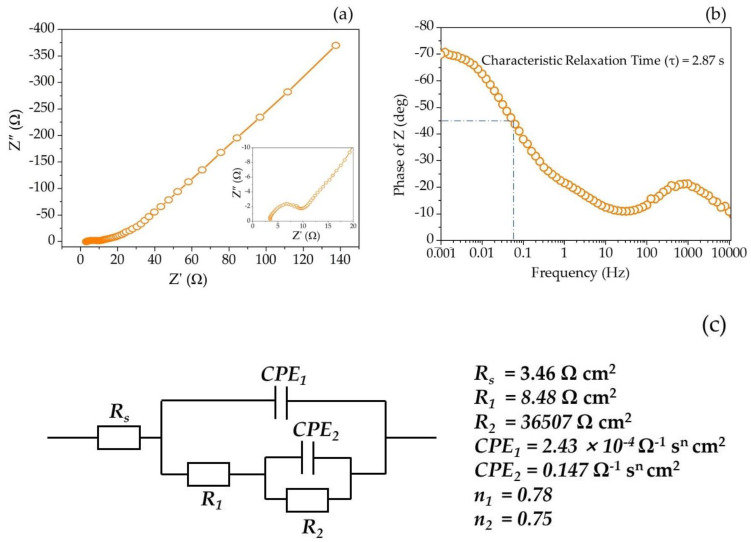
(**a**) Nyquist plot; (**b**) Bode plot depicting the material’s response time (characteristic relaxation time); (**c**) the electrical equivalent circuit to fit the impedance data obtained for the SPani-AC composite and the calculated circuit elements.

The internal resistance and capacitive characteristic of the SPani-AC electroactive material was studied using electrochemical impedance spectroscopy (EIS) and shown in Figure 8a,b. The Nyquist plot displays X-intercept in the high-frequency region, representing the electrolyte (1.0 M aqueous Na_2_SO_4_) resistance of 3.46 Ω cm^2^ (Figure 8a). The Bode plot displays material’s response time or characteristic relaxation time ‘τ’ of 2.87 s (Figure 8b). The calculated parameters obtained from the equivalent electrical circuit are shown in Figure 8c.

#### 2.2.5. Electrochemical Performance of Symmetric Device made of SPani-AC in Agar- Na_2_SO_4_ Polymer Gel Electrolyte

**Figure 9:**
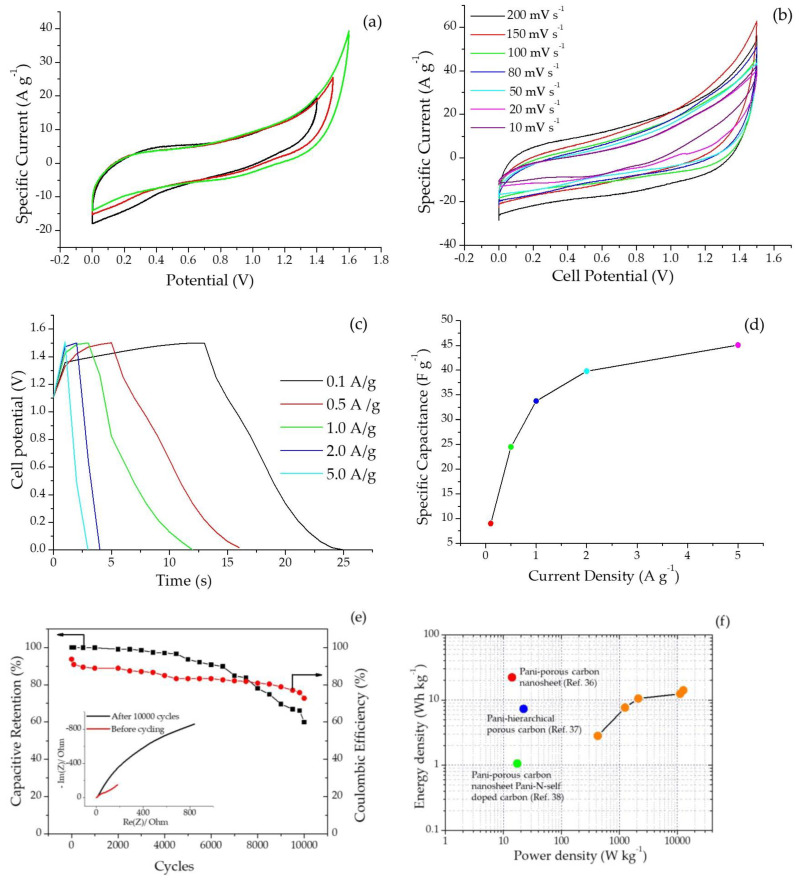
(**a**) Cyclic voltammograms recorded for a symmetric device using Agar-Na_2_SO_4_ polymer gel as an electrolyte in different voltage windows, ranging between 0.0 V and 1.4 V, 1.5 V, and 1.6 V, scanned at 50 mV s^−1^; (**b**) cyclic voltammograms at different scan rates, 10, 20, 50, 80, 100, 150, and 200 mV s^−1^; (**c**) GCD curves at different current densities; (**d**) evolution of specific capacitance with respect to the different current densities; (**e**) long-term cycling stability and Coulombic efficiency up to 10,000 continuous charge–discharge cycles at a current density of 0.1 A g^−1^, and Nyquist plots recorded before and after the cyclic stability test; (**f**) energy and power density as functions of the current density of the symmetric device assembly.

A symmetric device made of two electrodes loaded with SPani-AC composite matrix was tested for its electrochemical performance and cyclic stability using Agar-Na_2_SO_4_ polymer gel as an electrolyte. The cyclic voltammograms in different voltage windows, ranging between 0.0 V and 1.4 V, 1.5 V, and 1.6 V, scanned at 50 mV s^−1^ are shown in Figure 9a. Cyclic voltammograms scanned at different scan rates, viz., 10, 20, 50, 80, 100, 150, and 200 mV s^−1^ are shown in Figure 9b. GCD curves recorded at different current densities, viz., 0.1, 0.5, 1.0, 2.0, and 5.0 A g^−1^ are shown in Figure 9c. Th evolution of specific capacitances as a function of current density has been shown in Figure 9d. Long-term cycling stability and Coulombic efficiency up to 10,000 continuous charge–discharge cycles at a current density of 0.1 A g^−1^ are displayed in Figure 9e, and Nyquist plots recorded before and after cyclic stability test are shown in the inset of Figure 9e. Ragone plots of the APani-AC symmetric device assembly are shown in Figure 9f.

For calculating the specific capacitance of individual electrodes in a symmetric cell measured in the two-electrode configuration, the following equation is employed:

*C_sp_*= 4 × (*i* · Δ*t*)/(*m* · Δ*V*) (8)

where ‘*i*’ is current, m = total mass of the active material deposited on the two electrodes, ‘Δ*t*’ is the discharge time (s), and ‘Δ*V*’ is the potential window. The device exhibits a specific capacitance of 45 F g^−1^ at a current density of 5 A g^−1^, and the specific capacitances appear in an increasing order as the current density increases. This means that as the supply of electrons increases in the matrix, the latter acquires an increased capacity to accommodate the surplus charges. This pattern is the same as it appears in the case of three-electrode configuration. The specific capacitance calculated from the GCD curves recorded from a symmetric cell using Agar-Na_2_SO_4_ polymer gel as an electrolyte is 9 F g^−1^, which increases up to 45 F g^−1^ at 5.0 A g^−1^, showing an increase of 400% compared with the initial capacitance. This phenomenon is a unique feature exhibited by the material and can be explained by combining the theories of electrical double-layer charge storage and quantum chemistry (discussed in detail in the discussion section of this manuscript). Polyaniline-porous carbon nanosheet composite has been reported to exhibit energy and power densities of 22.3 KW kg^−1^ and 14.0 Wh kg^−1^, respectively [36]. On the other hand, polyaniline in a composite structure with hierarchical porous carbon has been reported to deliver energy and power densities of 17.3 KW kg^−1^ and 1.06 Wh kg^−1^, respectively [37]. In another report, polyaniline composite with an N-self doped carbon framework has been reported to deliver energy and power densities of 22.2 KW kg^−1^ and 7.31 Wh kg^−1^, respectively [38]. The Coulombic efficiency is calculated from equation 9 as follows:

(9) $$Coulombic\;efficiency=\frac{△t_{discharge}}{△t_{charge}}\times 100$$

Long-term cycling stability, specific capacitance retention, and Coulombic efficiency up to 10,000 continuous charge–discharge cycles at a current density of 0.1 A g^−1^ are shown in Figure 9e. The capacitive retention calculated was found to be 92.5%, and the Coulombic retention was found to be 98.8%. The energy density (E_sp,_ Wh kg^−1^) of the SPani-AC electroactive material is calculated using the following equation:

(10) $$E_{sp}=\frac{1}{2}C_{sp}{\left(△V\right)}^{2}$$

where ‘*C_sp_*’ is the specific capacitance (F g^−1^) and ‘Δ*V*’ is the working potential range. The power density (P_sp_, kW kg^−1^) of the SPani-AC electroactive material is calculated from Equation (11),

(11) $$P_{sp}=\frac{E_{sp}}{△t}\times \frac{3600}{1000}$$

where ‘*E_sp_*’ is the energy density (Wh kg^−1^) and ‘Δ*t*’ is the time elapsed during discharge process. The Ragone plot, or a plot of energy density vs. power density, is shown in Figure 9f.

## 3. Discussion 

### 3.1. Electrochemical Behavior of SPani-AC

The CV curves appear quasi-rectangular in shape, suggesting signature surface-redox pseudocapacitive behavior. The curves appear entirely different from the CV curves reported elsewhere for pristine Pani and do not show sharp redox peaks. This is attributed to the change in current response arising from the modification of surface micro-architecture due to inclusion of carbon particles in the composite. The area enclosed in the CV curves appear in an increasing order with an increase of scan rates. The CV curves recorded at higher scan rates display diminished or no redox peaks, indicating the charging current outrunning the rate of the redox reactions which occur at the surface of the electroactive material. In addition, at faster scan rates, the dimension of the diffusion layer formed at the interface of the electrode–electrolyte interface shrinks. This renders an increase in the flux of ions in the electrolyte to reach at the surface of the electroactive material supported on the electrode, and the latter results in a greater current response at higher scan rates [6,13,15,39,40]. The calculated specific capacitances display a decreasing trend with an increase in the scan rate. This occurs due to the fast movement of the ions in the electrolyte which deprives the ions from spending sufficient time at the electrode surface and penetrating deeply into the folds of the electroactive material, eventually resulting in fewer charges at the interface, resulting in low specific capacitances [35]. The GCD curves display an apparent deviation from the ideal triangular curve characteristic of capacitors. This is attributed to a pseudocapacitive charge–storage mechanism occurring in the material, which involves both charge storage with the formation of an electrical double layer and redox reactions occurring in the polyaniline molecule. In addition to this, there also appears to be a substantial decrease in the charging and discharging time with an increase in the current density. This can be explained based on events occurring at the electrode-electrolyte interface. At higher current densities, the ions present in the electrolyte do not have sufficient time to invade the deeper folds of the electroactive material supported on the working electrode and are restricted to a limited interaction with a fewer number of sites on the surface of the electroactive material to deliver a quicker charge–discharge process. The specific capacitances calculated from gravimetric charge–discharge curves present an increasing trend with an increase in the current density. This suggests a possibility of higher capacity for charge storage when the current is increased. The capacitive retention calculated for the two-electrode symmetric electrochemical cell was found to be 96% up to continuous charge–discharge 4500 cycles and observed to gradually decline at the end of 10,000 cycles. On the other hand, Coulombic efficiency was observed to be retained up to 85% until 4500 continuous charge–discharge cycles, declining up to 72% at the end of 10,000 cycles. This unusual property is explained by a combination of theories, namely microstructural and morphological properties of the material, interrelating molecular structures, and quantum chemical aspects related to the polyaniline molecule and corresponding polymer chain (discussed in detail in the next section).

The electrochemical behavior exhibited by an electroactive material is investigated by means of (a) the response to scan rates in cyclic voltammetry; (b) the response to constant current in GCD measurements; and (c) the response to the AC current in the electrochemical impedance measurements. The current responses obtained from the CV measurements at different scan rates allow differentiating the diffusion-controlled process and non-diffusion limited processes quantitatively [13,41,42]. The total charge stored by an electroactive material is a sum of two processes, viz., (a) redox reactions and (b) capacitive processes. In the case of SPani-AC, the redox reactions involve diffusion intercalation of ionic species and change in the conformation of the polymer molecule. The capacitive process involves the formation of electrical double layer charge storage and pseudocapacitance. The value of ‘*b*‘ can predict the predominating charge storage mechanism occurring in the electroactive material, whether it is intercalative, capacitive, or pseudocapacitive. A ‘*b*’ value of 1.0 indicates a dominance of fast surface-controlled reactions associated with adsorption–desorption of the electrolyte ions, whereas the ‘*b*’ 0.5 indicates diffusion-controlled redox processes. The ‘*b*’ value ranging between 0.5 and 1.0 indicates a transition from battery-type behavior to capacitive behavior. To obtain the signature electrochemical characteristic features in a broader range of scan rates and current densities, and a better fit of the Power law, the CV curves are required to be recorded at relatively slower scan rates (~1–5 mV s^−1^) to evaluate ‘*b*’ values using Dunn’s method. The CV curves recorded at swifter scan rates exhibit a significant shift of redox peaks [13]. The ‘*b*’ values help in understanding the electrode kinetics and the charge storage mechanism occurring in the electroactive material. Further, the analysis of electrochemical processes is assisted with AC impedance results. 

### 3.2. Formation of SPani-AC and Role of Molecular Structure and Morphological Properties on Its Electrochemical Behavior

Polyaniline is formed from oxidative polymerization of aniline in acidic medium. The reaction is an exothermic process. In the present study, the polymerization of aniline was carried out by mixing two hot and dilute solutions, namely aniline in 1 N HCl containing dispersed activated carbon particles in the medium and ammonium peroxydisulfate in 1 N HCl (oxidant) at 60 °C. The elevated temperature slows down the rate of the polymerization reaction. The aniline molecules, when dispersed in the solution containing activated carbon particles, get adsorbed on their surfaces. The carbon particles act as nucleation sites for the aniline polymerization reaction. When oxidant molecules are added to the reaction mixture, the peroxydisulfate ions, being one of the most efficient electron acceptors, initiate oxidative polymerization of aniline. The process begins with a two-electron oxidation mechanism to form aniline nitrenium cation [43]. The progress of aniline polymerization takes place in three main steps: initiation, propagation, and termination. In the present study, the synthesis of SPani-AC was carried out at an elevated temperature of 60° which is believed to slow down the reaction kinetics, as polymerization of aniline is an exothermic reaction. Furthermore, the addition of oxidant was carried out at a very slow rate, drop by drop to allow the molecules to have sufficient time to reorganize themselves in stable equilibrium molecular geometries via bond rotation at ‘N-C’. As the chain length grows, the aniline dimer grows into trimer, oligomer, and consequently a polymer (Pani). The pH of the reaction did not undergo any change, therefore implying that the chain propagation occurred predominantly linearly, and the formation of branching or intermediates could be ruled out. It is reported that the branching in polyaniline chain is witnessed when the polymerization reaction is carried out at a pH >2.5. Intermediates, such as benzidine (20% to 50%), are formed below the pH range of 1.0 and −2.0, whereas the formation of hydrazobenzine and azobenzine takes place at a higher pH >1.0 [44,45,46,47]. 

**Figure 10:**
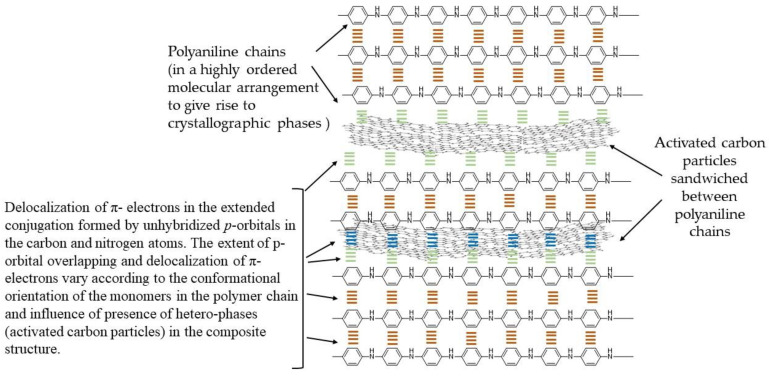
Polyaniline-activated carbon-sandwiched structure. The orderly arranged polyaniline chains give rise to well-defined crystallographic phases. The activated carbon particles are sandwiched between the long polymer chains.

The nitrogen atom in the aniline monomer and the terminal nitrogen atoms in the growing chains of the polyaniline act as oxidation centers. The terminal nitrogen atoms possess a lower oxidation potential and are therefore activated preferentially over other nitrogen atoms in the interiors of the polymer molecule, making the remaining part of the polymer behave as dormant. At every addition of a monomer (nitrenium cation) to the growing polymer chain, a release of two protons occurs, which causes a dip in the pH of the solution. Furthermore, the very slow addition of the two reactant solutions did not cause any noticeable change in pH in the present study. As the polymerization progresses at different nucleation sites, or as carbon particles are dispersed in the reaction medium, and at independent nucleation sites (with no carbon particles associated or present in the vicinity), the polyaniline chains that grow in chain length as well as in numbers begin to interact with one another. The neighboring chains interact to form pπ-pπ bonding between the unhybridized *p*-orbitals present in the carbon and the nitrogen atoms. A very large number of such interactions between the neighboring polyaniline chains give rise to highly ordered and well-defined crystallographic lattices, as shown in HR-TEM images in Figure 2. As the polymerization further progresses, the polyaniline chains coil and supercoil to form fiber- or spindle-shaped structures [15]. The supercoiling of polyaniline chains into spindle-shaped entities cause detachment from the carbon particle surfaces, and the two different species in the matrix therefore acquire separate boundaries. However, the polyaniline spindles exist as closely associated and interwoven with one another, as shown in the SEM images in Figure 1. The carbon particles are randomly scattered in the polyaniline matrix and are believed to introduce porosity. The formation of semi-polycrystalline phases in SPani-AC sandwiching activated carbon particles is explained in the Figure 10. 

### 3.3. Electrochemical Impedance Spectrum Analysis

The impedance response by an electroactive material reveals resistive and capacitive properties of the charged layer formed on its surface. The Nyquist plot (Figure 8a) displays two depressed semicircular arcs. The small value of R_s_ value indicates that the electrolyte is feasible for achieving more intercalation−deintercalation reaction with the SPani-AC electroactive material. In addition, two depressed semicircular arcs in medium- and low-frequency regions represent charge transfer resistance at the electrode−electrolyte interface and the diffusion process related to the morphology of the electrodes [48]. In the Bode plot (Figure 8b), there are two apparent humps indicative of two *CPE* components. In the Bode plot, the lower frequency phase angle approaches closer to −90°, indicating that the electroactive material is performing similar to a ‘capacitor-type’ material. The small semicircle appears in the high-frequency region, and the large semicircle appears in the lower frequency region. The shapes of the two semicircles are outcomes of a combination of factors, e.g., the structure and properties of the charged layer on the electrode surface. The depressed semicircles (the center of the circles lie below the axis) indicate deviation from ideal capacitive behavior. Therefore, the capacitance arising due to the formation of an ideal double layer, or ‘*C_dl_*’, is replaced by ‘*CPE*s’, or ‘constant phase element’. The latter is a non-intuitive circuit element included in the electrical equivalent circuit to explain the response of a real system that shows deviation from ideal behavior. On the other hand, in an ideal case, the semicircular capacitive arcs have their centers lying on the *x*-axis of the Nyquist plot. The deviation from the ideal behavior is attributed to the interaction of the electroactive material’s surface and the electrolyte, surface roughness, or surface inhomogeneity. The latter defines the distribution of the reaction rates of the redox reactions at the active sites with varying energies present on the surface of the electroactive material. *CPE*_1_ represents double layer, and the combination of ‘*CPE*_2′_, ‘*R*_1′_, and ‘*R*_2′_ in parallel with *CPE*_1_ represents pseudocapacitance. ‘*R*_1′_ represents non-capacitive resistance associated with redox reactions, and ‘*R*_2′_ represents non-capacitive resistance associated with discharge or desorption of adsorbed species. In the case where the redox reactions are truly Faradaic (involving a phase change in the electroactive material), the two resistances, ‘*R*_1′_ and ‘*R*_2′_, are termed Faradaic resistances. ‘*R*_1′_ is precisely termed Faradaic resistance associated with redox reactions that are irreversible. ‘*R*_2′_ represents Faradaic resistance associated with discharge (desorption of adsorbed species) [18,49]. 

The *CPE* is mathematically defined as

(12) $$Z_{CPE}=A^{-1}{\left(i\omega \right)}^{-n}$$

where, ‘*Z_CPE_*’ is *CPE*’s impedance and ‘*A*’ is the *CPE* parameter prefactor. The latter is defined as admittance (*1/*|*Z*|*)* at an angular frequency ‘*ω*’ rad s^−1^ (Ω^−1^ cm^−1^ s^n^). The symbol ‘*i*’ is an imaginary number defined as (*i = √-1*). According to this simple equation, the impedance measurement allows the phase angle of the *Z_CPE_* to be independent of the frequency and holds a value of (*−n × π/2*) degrees. Hence, the *CPE* is denoted by this name. The factor ‘*n*’ is the inhomogeneity coefficient, and the inhomogeneity in an electroactive material arises due to porosity. In the present study, active carbon is introduced into polyaniline during the synthesis process, which is believed to have created porous features in the material. The value of *n* =1.0 is indicative of an electroactive material behaving as an ideal capacitor, whereas a deviation from unity (i.e., ‘*n*’ values lower than unity) suggests a deviation from ideal capacitive behavior and a shift towards diffusion-controlled redox behavior. 

In the present study, the two ‘*n*’ values corresponding to the two *CPE*s in the electrical equivalent circuit are 0.78 and 0.75, respectively. The ‘*n*’ values calculated for the two *CPE*s (*CPE*_1_ and *CPE*_2_) for semi-polycrystalline polyaniline electroactive material in our previously reported work was 0.80 and 0.82, respectively. The decrease in the ‘*n*’ values indicate relatively higher inhomogeneity in the SPani-AC composite compared to pristine polyaniline (Pani), which is attributed to the introduction of activated carbon in the material architecture. However, the ‘*n*’ values are close to unity, indicating a reasonable dominance of capacitance and a relatively greater share of the diffusion-controlled process compared to pristine Pani from the previously reported work. The determination of the elements in the equivalent electrical circuit was carried out by fitting the measured data using a simulation program; a detailed description and the usual meaning of the elements in impedance analysis are explained in detail elsewhere [15]. The electrical equivalent circuit and the calculated circuit elements, viz., *R*_s_ (solution resistance), *R*_1_, and *R*_2_ (resistances corresponding to charge transfer and diffusion processes, respectively), *CPE*_1_ and *CPE*_2_ (constant phase elements corresponding to double-layered capacitance and pseudocapacitance, respectively) are shown in Figure 8c. The development of a supercapacitor for real-life practical applications is characterized by rapid power delivery and long cyclic performance. To estimate the merit of the supercapacitor material’s response time or characteristic relaxation time, the ‘τ’ value is estimated from Bode phase angle vs. frequency plot. The frequency (*f*) value is determined from the phase angle approaching −45°, the characteristic point where both real and imaginary impedances equal each other. In the present study, the ‘*τ*’ value is 2.87, suggesting that the SPani-AC-based supercapacitor electrode can handle and deliver high throughput-rate power. The Nyquist plots obtained from the impedance measurements before and after cyclic tests in the case of two-electrode symmetric device (shown in the inset of Figure 10e) reveal ‘R^s^’ of 0.17 Ω, indicating greater concentration of ions in the electrolyte phase available to the active material in comparison to the aqueous electrolyte. The shapes of the semicircular arc after cyclic stability test indicate capacitive features.

### 3.4. Application of Molecular Orbital (MO) Theory for Explaining the Charge Storage Mechanism in SPani-AC Composite Electroactive Material

**Figure 11:**
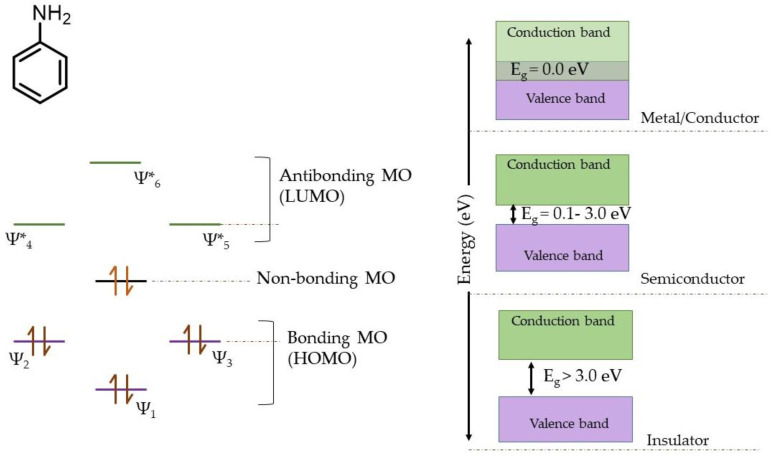
MO diagram of π-orbitals in aniline molecules and the overlapping of energy bands in a conducting polymeric material (polyaniline).

For a conductive polymer to act as a conductor, semiconductor, or an insulator, it is crucial to possess an appropriately suitable molecular structure, particularly uniformity of the conjugation which alternately implies existence of an extended π-electron cloud. Conductivity and charge storage properties both can be explained by using molecular orbital (MO) theory and band theory in the context of the quantum chemical theory of atomic structures. The atomic orbitals combine to give rise to an equivalent number of MOs, namely bonding MOs (with lower energy states) and anti-bonding MOs (with higher energy states). The MOs somewhat resemble their corresponding parent atomic orbitals, and the electrons belonging to the atomic orbitals are redistributed in MOs. The MO diagram of π-orbitals in aniline molecules and overlapping of energy bands in a conducting polymeric material (polyaniline) is shown in Figure 11.

The MOs are further classified into HOMO (highest occupied molecular orbital) and LUMO (lowest unoccupied molecular orbital). In a crystalline solid or a metal (conductor), the atoms are arranged in a systematic lattice and chemically bonded. The electrons on a particular atom experience a shared influence of the electrical field due to the presence of electrons from other atoms, and the electrons can change their positions to other atoms, consequently broadening the atomic energy states (which are sharp in individual atomic orbitals), and thus ‘energy bands’ form in the solid [15,50,51]. There are six π-electrons from six carbon atoms in the aromatic ring of the aniline molecule and one lone pair of electrons on the nitrogen atom of the amine group, altogether eight π-electrons participating in resonance. Consequently, the seven *p*-orbitals belonging to six carbon atoms and one nitrogen atom combine to form seven molecular orbitals, namely *ψ*_1_, *ψ*_2_, and *ψ*_3_ as bonding MOs associated with lower energy states, and ψ*_1_, ψ*_2_, and ψ*_3_ as anti-bonding MOs associated with higher states, and a non-bonding MO in the barycenter. The eight π-electrons are accommodated in order of lower to higher energy states to fill bonding and anti-bonding MOs. Accordingly, all bonding MOs and the non-bonding MO are filled, and the anti-bonding MOs remain vacant. Consequently, when a very large number of atomic orbitals (or ‘*N*’, ~10^22^ for a 1 cm^3^ of material matrix) possessing equivalent energies interact to give rise to ‘*N*’ number of Mos, such a significant number of bonding and anti-bonding MOs together construct ‘valance’ and ‘conduction’ bands, respectively. The difference between the two is called ‘band gap’ or ‘*E_g_*’, and the energy states associated with the band gap are not available to the electrons. In the conducting material, the energies of valence and conduction bands overlap, allowing delocalization and the fast transition of electrons. For highly ordered crystalline phases in a material, such as pristine Pani [15] or SPani-AC in the present study, the overlapping of valence and conduction bands facilitate delocalization of electrons and render the material conducting. It is apparent from the MO diagram that the anti-bonding MOs, being vacant, can accommodate extra electrons. Consequently, the conduction band formed from a huge number of LUMOs possess ample energy states for accommodating extra electrons conveniently entering during the charging process in GCD measurements as the current density is increased [15,52].

### 3.5. Mechanism of Charge Storage in SPani-AC

The SPani-AC composite material possesses randomly scattered nanostructured patches of well-defined crystallographic lattices, as seen in HR-TEM images. These phases support structural build-up of an electrochemical double layer conducive of capacitor-type behavior. This feature is missing in the amorphous materials, where the ion-diffusion mechanism dominates, e.g., in Pani-based electroactive materials synthesized by conventional methods where molecules do not form crystallographic lattices. During the electrochemical tests, viz., CV and GCD in the Na_2_SO_4_ electrolyte, the mechanism of charge storage can be explained as follows: the Na_2_SO_4_ dissociates to release Na^+^ and SO_4_^2-^ ions into the aqueous medium. The ions become hydrated or surrounded by water molecules and form hydration sphere(s). The oxygen and the hydrogen atoms in the water molecules of the hydration sphere acquire partial charges during the charging process, when the ions drift towards the respective electrodes along with their hydration spheres, and the structural build-up of double layer takes place at the electrode-electrolyte interface. As explained in our previous works [15,52], the terminal ends of the Pani chains are the active sites for redox reactions to occur in the molecule. Furthermore, the redox reactions occurring in the Pani molecule do not lead to any phase change of the material. Rather, conformational change occurs in the molecule, and the process is highly reversible. Hence, the redox reactions occurring in Pani molecules are completely non-Faradaic. Faradaic reactions involve a phase change in materials upon consumption of electrons, and the process is highly irreversible. It is important to note that according to Conway definitions, SPani-AC cannot be denoted as a battery material; rather, it is denoted as a ‘redox capacitor’. Inner portions of the polymer molecule remaining embedded in supercoiled architecture are stabilized by geometry, morphology, and resonance due to π-orbital overlapping, intermolecular H-bonding, and van der Waals forces of attraction and resist any change in molecular conformation brought upon by redox reactions. The dormancy of the Pani inner architecture provides a stable surface on which to support the structural build-up of an electrochemical double layer. It is believed that the change in the molecular conformation during the redox reactions is a reason for a deviation from ideal signature features of a capacitor and the appearance of pseudocapacitance. Precisely, the pseudocapacitance in SPani-AC material arises due to surface quinonoid reactions and partial charge transfer during electrosorption of charged species at the N-sites of the Pani, resulting in oxidation and conformational change in the polymer chain at the respective sites. The presence of carbon particles in the SPani-AC material introduces porosity in the material, which is believed to expose more Pani terminal sites for redox reactions. It is therefore argued that the material in the present study, SPani-AC, for which the redox reactions are largely restricted at the surface of the material, it is termed a ‘surface-redox supercapacitor’ material [14,15,53].

## 4. Experimental

### 4.1. Materials

All the reagents used for synthesis, namely aniline (99.9%), activated carbon (mesoporous, and BET surface area of 162 m^2^ g^−1^), and N-methyl 2-pyrrolidone (NMP) (99.5%), were procured from Sigma-Aldrich, Republic of Korea; ammonium persulfate (98.9%), sodium sulphate dibasic (99.0%), hydrochloric acid (37.0%), sulfuric acid (95.0%), methanol (99.5% by vol.), and ethanol (94.0% by vol.) were procured from Duksan Pure Chemicals Co. Ltd., Republic of Korea. The activated carbon used in this study was a commercial product with a surface area of 162 m^2^ g^−1^ (reported in the product specification)_._ The BET surface area for Polyaniline-AC composite was analyzed experimentally to be 34 m^2^ g^−1^. The chemicals were utilized as received. 

### 4.2. Synthesis of Semi-Polycrystalline Polyaniline-Activated Carbon Composite

**Figure 12:**
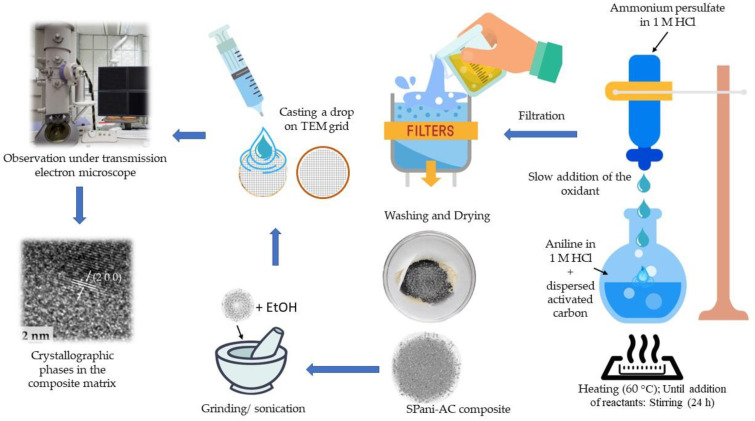
Schematic explaining synthesis of SPani-AC composite.

The SPani-AC composite was prepared using in-situ oxidative polymerization of aniline in an aqueous acidic medium. Sequentially, 1.0 g of activated carbon was dispersed in 300 mL of 1 N HCl sonicated to obtain a homogenous suspension of carbon particles in the aqueous medium. The solution was then heated to 60 °C in a round-bottomed flask fitted with a reflux condenser using an oil bath and constantly stirred using a magnetic stirrer. Another solution was prepared by dissolving 7.4 g of ammonium persulfate in 200 mL of 1 N HCl and heated separately up to 60 °C. The two hot and diluted solutions were then mixed slowly by adding ammonium persulfate solution drop by drop to the reaction medium. The temperature of the solution was kept at 60 °C throughout the addition of the oxidant. As the addition of the oxidant progressed, the color of the solution gradually turned dark green due to the polymerization of aniline and the subsequent precipitation. As the polymer chain length grows, it starts coiling and supercoiling to engulf tiny carbon particles in its folds, resulting in the formation of composite. The reaction mixture was continually stirred at 60 °C for 2 h after completion of the oxidant addition. The heating was then stopped, and the reaction mixture was allowed to cool by itself and was continually stirred for the next 14 h. After the stirring was stopped, the precipitates settled at the bottom of the round-bottomed flask due to gravity, and the supernatant solution appeared transparently clean. The supernatant solution was then decantated and precipitate was filtered using a Buckner funnel fitted with a Whatman filter paper, washed with deionized water and methanol, respectively. The precipitate in the filter paper was then washed with 500 mL of 1 M NH_4_OH and kept overnight, followed by washing with 2 L of deionized water and 200 mL of MeOH, respectively. After filtration and washing, the precipitate was allowed to dry overnight at room temperature in air and then transferred to a glass petri dish and dried at 70 °C in an electric oven overnight and stored in a glass vial. A schematic explaining the synthesis of SPani-AC composite is shown in Figure 12.

### 4.3. Material Characterization

The microstructure and phase of the newly synthesized semi-polycrystalline polyaniline-activated carbon (SPani-AC) composite were characterized using a scanning electron microscope, a field-emission scanning electron microscope (FE-SEM) (HITACHI-S4800), operating at an accelerating voltage of 5 kV in a vacuum of 10^−4^–10^−6^ mm Hg; a field emission transmission electron microscope (FE-TEM, FEI, Tecnai G20, TWIN, USA), operating at an accelerating voltage of 200 kV; and X-ray diffraction (XRD PANalytical, X-pert PRO MPD, the Netherlands), respectively. For recording high-resolution TEM images (HRTEM), a very fine dispersion of the composite material was freshly prepared. Sequentially, a small amount of the dried powder (0.5 g) was placed in an agate mortar, and ground using excess ethanol (5 mL) until the entire consistency became homogeneous. It is believed that the freshly synthesized composite material is accumulating impurities, e.g., unreacted solvent/reactant molecules in its folds, which needs to be removed to capture good images. Hence, the material was washed accordingly. The insoluble content (composite material) was allowed to settle in the excess ethanol, and the supernatant was removed. The settled particles were again ground in excess ethanol, allowed to settle, and the supernatant was removed. The steps were repeated 3–4 times, and finally a fine suspension in 1 mL of ethanol was preserved for imaging. In the final stage, a drop of the solution from this suspension was cast onto a copper grid using a syringe and dried. The specimens were now ready for imaging using HR-TEM. The XRD spectrum was recorded by scanning the polyaniline samples in the 2*θ* range from 10° to 80° using the X-ray diffractometer equipped with Cu-Kα radiation (λ = 0.154 nm). Laser Raman spectra were recoded using LabRam HR800 UV Raman microscope (Horiba Jobin-Yvon, France KBSI) with a laser beam (λ = 532 nm). 

### 4.4. Electrochemical Characterization

The electrochemical experiments, viz., cyclic voltammetry, galvanostatic charge–discharge (GCD), and electrochemical impedance spectroscopy were carried out using an electrochemical analyzer (Potentiostat, VersaSTAT-3, Princeton Research, USA). All the electrochemical measurements were performed at room temperature using an electrochemical cell with a three-electrode configuration. The electrolyte used in the experiments was 1.0 M aqueous Na_2_SO_4_. The working electrodes were fabricated by drop casting polyaniline-activated carbon slurry on nickel foam substrate within an area of 1.0 cm^2^. To make the slurry, polyaniline-activated carbon composite powder (weighing 6 mg), polyvinylidene difluoride (PVDF) (2 mg), and carbon black (2 mg) were mixed with 2 mL of N-Methyl-2-pyrrolidone (NMP) solvent using a miniature agate mortar. The drop casting of the slurry was carried out by pipetting out 100 μL of the slurry using a micropipette and casting it over Ni-foam of a dimension of 3 cm × 1 cm within the pre-defined area of 1.0 cm × 1.0 cm, followed by drying in an oven at 60 °C overnight. The weight of the transferred material (polyaniline-activated carbon composite slurry or electro-active material with binder) was calculated by subtracting the weight of the Ni-foam. The active material-loaded Ni-foam was utilized as working electrode. The other two electrodes, viz., the counter electrode and the reference electrode, were platinum sheets of 1 cm^2^ and Ag/AgCl in 3 M KCl, respectively. The electrochemical responses corresponding to cyclic voltammetry were recorded at different scan rates ranging from 10 mV s^−1^ to 1 V s^−1^ in the potential range of −0.6 V to 0.8 V. EIS measurement was performed in a frequency range of 100 kHz and 0.1 Hz with the application of an AC perturbation of 5 mV. The galvanostatic charge–discharge (GCD) curves were recorded at different current densities ranging from 1 to 15 A g^−1^.

In order to explore the performance of SPani-AC composite material in a two-electrode set-up, a symmetric device was assembled by depositing the active material on the two pieces of nickel foam (with a dimension of 3 cm × 1 cm, as a current collector) incorporating Agar-Na_2_SO_4_ polymer gel in an aqueous medium as an electrolyte. The total active mass loading onto the two Ni-foam electrodes was 17.7 mg. The gel polymer electrolyte was prepared by dissolving agar agar and Na_2_SO_4_ in deionized water in a ratio of 1:1 (weight was calculated according to 1 M Na_2_SO_4_) and stirring the mixture solution at a temperature of 95–100 °C. Soon after the agar dissolved, the mixture was allowed to cool down and settle until gel formation initiated. The electrodes were then immersed into the gel with a glass fiber filter paper sandwiched between the two electrodes as a separator. The electrochemical characterization of the symmetric device was carried out using an electrochemical analyzer (Biologic multichannel VM3).

## 5. Conclusions

Semi-polycrystalline polyaniline-activated carbon (SPani-AC) composite material was synthesized by adopting an in-situ oxidative polymerization of aniline on the carbon surface in aqueous HCl at an elevated temperature of 60 °C. The specific capacitance calculated from CV and GCD curves is 507 F g^−1^ at the scan rate of 10 mV s^−1^ and 298 F g^−1^ at 15 A g^−1^. The symmetrical two-electrode device exhibits a specific capacitance of 45 F g^−1^ at a current density of 5 A g^−1^. The capacitive retention calculated was found to be 96% up to 4500 continuous charge–discharge cycles and observed to be gradually declining at the end of 10,000 cycles. On the other hand, Coulombic efficiency was observed to be retained up to 85% until 4500 continuous charge–discharge cycles, which declines up to 72% at the end of 10,000 cycles, and EIS reveals dominance of capacitive behavior over ion-diffusion. Hence, SPani-AC presents a decent example of a surface redox supercapacitor electrode.

## Data Availability

N.M. acknowledges Professor Moo Hwan Cho (Retired), School of Chemical Engineering, Yeungnam University, Gyeongsansi, Gyeongsangbukdo, Republic of Korea-38541, for providing laboratory facilities for synthesis of the materials.
